# Author Correction: Barcoded HIV-1 reveals viral persistence driven by clonal proliferation and distinct epigenetic patterns

**DOI:** 10.1038/s41467-025-65523-3

**Published:** 2025-10-27

**Authors:** Tian-hao Zhang, Yuan Shi, Natalia L. Komarova, Dominik Wodarz, Matthew Kostelny, Alexander Gonzales, Izra Abbaali, Hongying Chen, Gabrielle Bresson-Tan, Melanie Dimapasoc, William Harvey, Christopher Oh, Camille Carmona, Christopher Seet, Yushen Du, Ren Sun, Jerome A. Zack, Jocelyn T. Kim

**Affiliations:** 1 Department of Molecular and Medical Pharmacology, University of California, Los Angeles, CA USA; 2 Molecular Biology Institute, University of California Los Angeles, Los Angeles, CA USA; 3 Department of Mathematics, University of California San Diego, La Jolla, CA USA; 4 Department of Ecology, Behavior and Evolution, University of California San Diego, La Jolla, CA USA; 5 Department of Microbiology, Immunology, and Molecular Genetics, University of California Los Angeles, Los Angeles, California, USA; 6 Department of Medicine, Division of Infectious Diseases, University of California Los Angeles, Los Angeles, California, 90095 USA; 7 Department of Medicine, Division of Hematology and Oncology, University of California Los Angeles, Los Angeles, California, USA; 8 Cancer Institute (Key Laboratory of Cancer Prevention and Intervention, China National Ministry of Education), The Second Affiliated Hospital, Zhejiang University School of Medicine, Hangzhou, Zhejiang China; 9 Center for Infectious Disease Research, School of Medicine, Westlake University, Hangzhou, Zhejiang China; 10 Present Address: Department of Molecular and Medical Pharmacology, University of California, Los Angeles, CA USA

**Keywords:** Virology, HIV infections, Viral reservoirs, Retrovirus

Correction to: *Nature Communications* 10.1038/s41467-025-56771-4, published online 14 February 2025

In this article, the author’s name Dominik Wodarz was incorrectly written as Dominik Wordaz. The original article has been corrected.

