# Supplementary material for: Barcoded HIV-1 reveals viral persistence driven by clonal proliferation and distinct epigenetic patterns
Source: Nat Commun. 2025 Feb 14;16:1641. doi: 10.1038/s41467-025-56771-4 (PMC11829055; doi:10.1038/s41467-025-56771-4)
Supplement: Supplementary file 1 — Supplementary Info [file 41467_2025_56771_MOESM1_ESM.pdf]

SUPPLEMENTARY INFORMATION

Barcoded HIV-1 reveals viral persistence driven by clonal proliferation and distinct epigenetic patterns

TABLE OF CONTENTS

Supplementary Figures: Fig. S1 to S12
Supplementary Note 1
Supplementary References

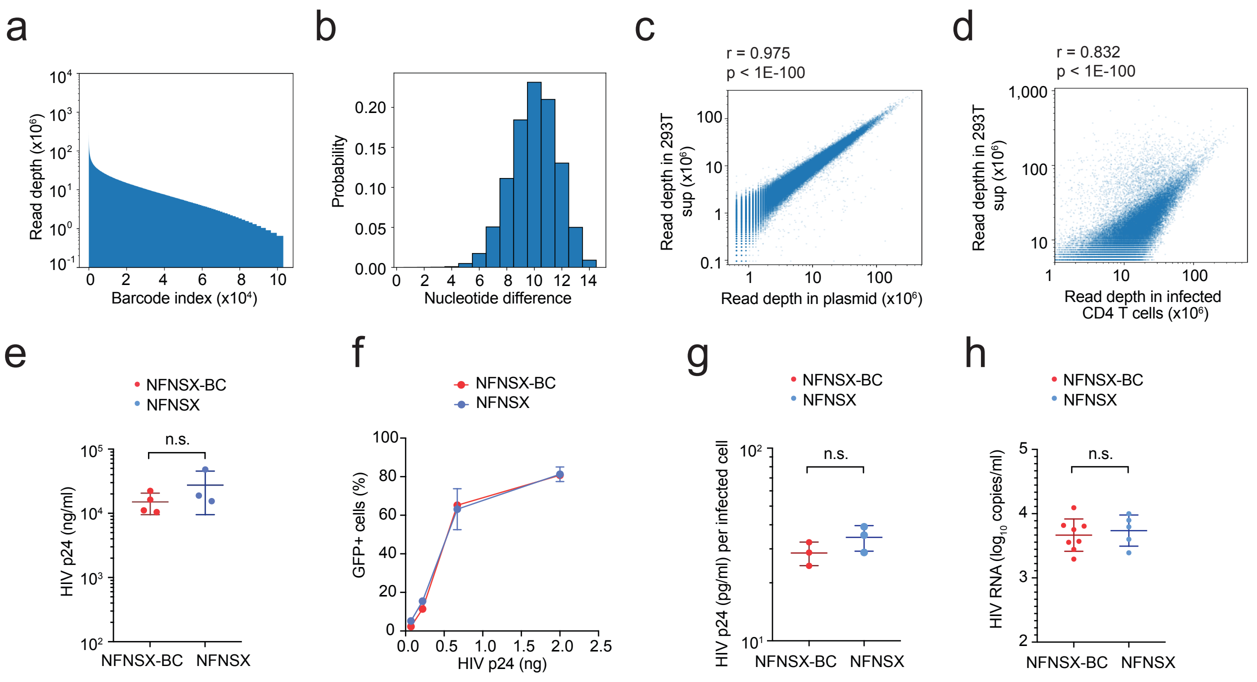

Fig. S1: Generation and in vitro infectivity of barcoded NFNSX.

a, Histogram of distribution of viral barcodes as quantified by deep sequencing derived from extracted viral RNA from the cell-free virus supernatant collected from virus-producing 293T cells transfected with NFNSX-BC plasmid library.

b, Histogram of the pair-wise distance between any two randomly selected viral RNA barcodes, indicating most viral barcodes were at least >3 nt and most commonly 10 nt in difference.

c, d, Scatterplot of the frequency of the viral barcodes in the plasmid library preparation versus the virus supernatant from transfected 293T cells (c) or the virus supernatant from 293T cells transfected with NFNSX-BC versus virus passaged in primary CD4+ T cells (d). r, Spearman's correlation coefficient with associated P value.

e, HIV p24 protein levels from the virus supernatant of 293T cells transfected with plasmids encoding NFNSX-BC or NFNSX was measured by ELISA. Data is mean ± SD. n= 3-4 biologically independent transfection preparations. P value was calculated using the two-tailed Mann-Whitney test. n.s., p > 0.05.

f, Varying input of NFNSX-BC or NFNSX virus supernatant was added to GHOST (3) CXCR4+CCR5+ cells. Infected GFP+ cells were quantified 48 h later by flow cytometry. Connecting lines indicate the mean ± SD. n = 3 technical replicates per group from each independent experiment. Data are representative of two independent experiments.

g, Production of HIV p24 from infected GHOST (3) CXCR4+CCR5+ cells. Equivalent dose of NFNSX-BC or NFNSX was used to infect GHOST (3) CXCR4+CCR5+ cells for 2 h. Infected cells were washed twice and then cell-free supernatant collected at 48 h. HIV p24 levels were measured over the number of infected GFP+ GHOST (3) CXCR4+CCR5+ cells. Data is mean ± SD. n = 3 technical replicates per group. P value was calculated using the two-tailed Mann-Whitney test.

h, Humanized mice were injected with 500ng p24 of NFNSX-BC or non-barcoded NFNSX. Plasma HIV RNA loads were measured by qRT-PCR 4 weeks after HIV injection. Data is mean ± SD. n = 8 mice per NFNSX-BC group. n = 5 mice per non-barcoded NFNSX group. P value was calculated using the two-tailed Mann-Whitney test.

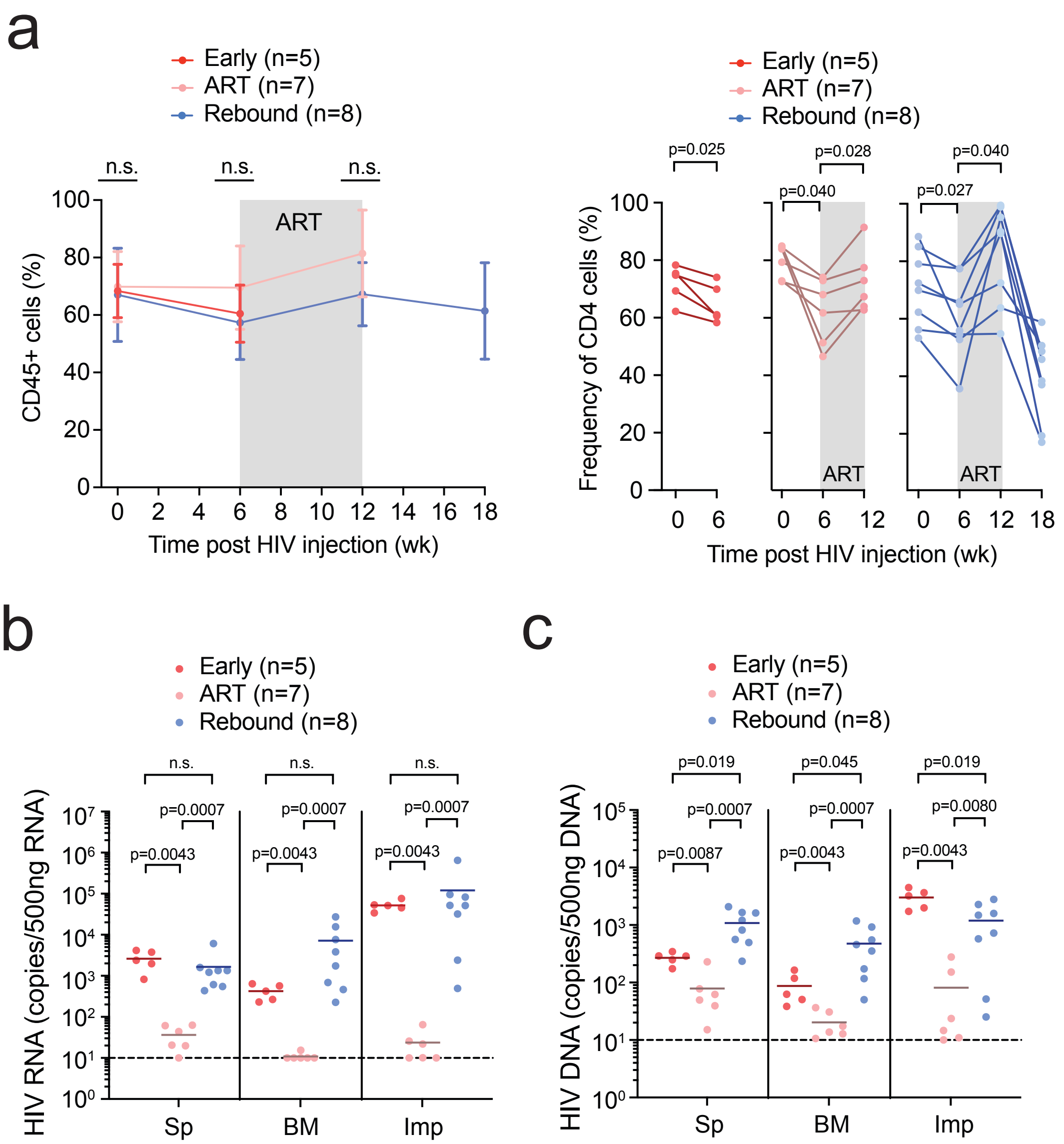

Fig. S2: Human immune engraftment and viral loads after NFNSX-BC infection in vivo.
a, Left, Longitudinal frequencies of human CD45+ cells. Gray shading indicates an ART treatment period. Mean ± SD. Frequencies of human CD45+ cells among the groups of mice was compared at time of HIV injection, early infection, and ART suppression. The p values were calculated using the one-way ANOVA test when 3 groups were compared, the p values were calculated using the two-tailed Mann-Whitney test. Right, Longitudinal frequencies of CD4+ T cells. Frequencies of human CD4+ T cells. The p values were calculated using paired t-test.
b, c, Cell-associated HIV RNA (b) and DNA (c) by RT-PCR and qPCR from the spleen (Sp), bone marrow (BM), and human thymic implants (Imp) of mice sacrificed at acute infection, ART suppression, and rebound infection. The black dashed line indicates the detection limit of 10 HIV RNA or DNA copies. Horizontal bars represent mean values. n.s., $p > 0.05$. P values were calculated using the two-tailed Mann-Whitney test.

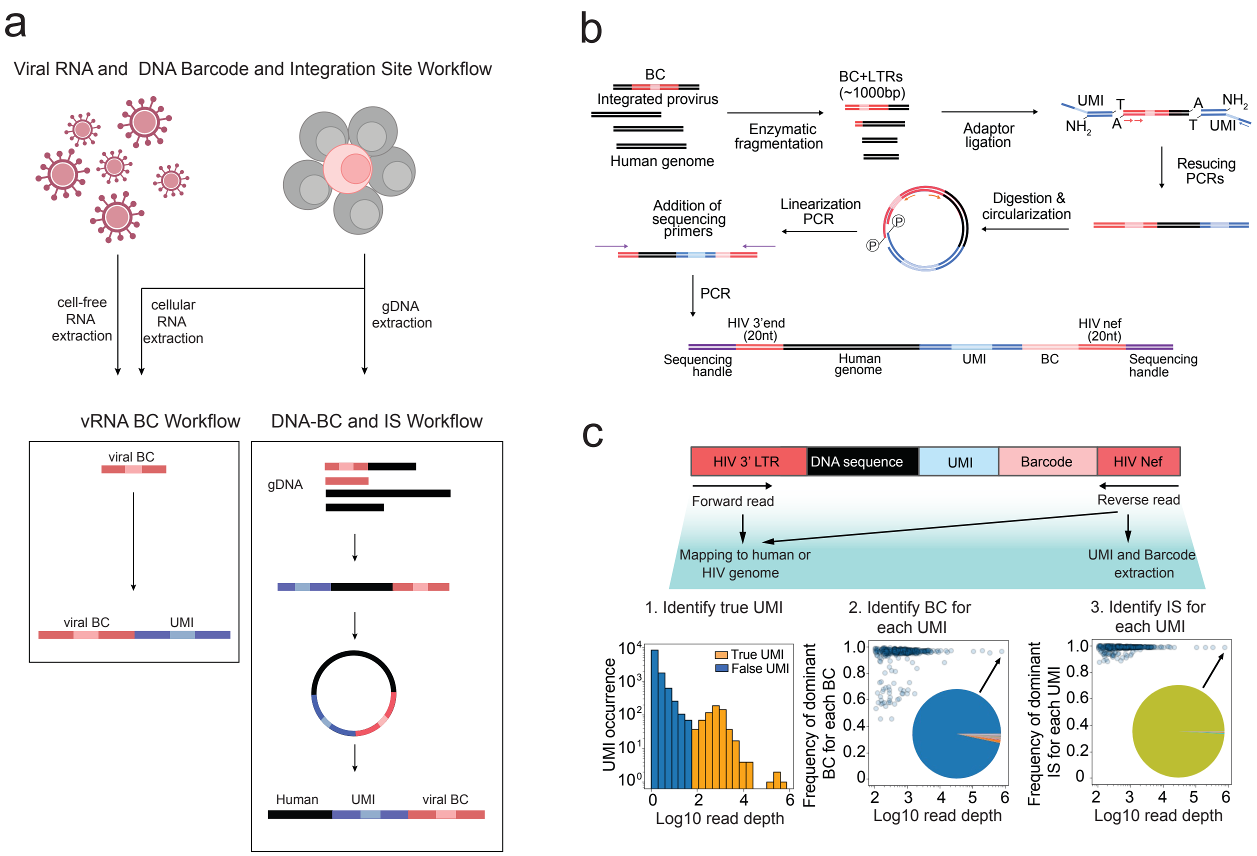

Fig. S3: Schematic of barcode workflow and viral DNA barcode linkage to integration site simultaneously via PCR.

a, Schematic depicting the overall experimental workflow to detect viral RNA barcodes and viral DNA barcode PCR linked to integration sites. Created in https://BioRender.com

b, Schematic delineating processing of genomic DNA for BI-seq.

c, Schematic depicting deep sequencing read, in which the forward reads contained the 3’ HIV LTR and if present the matching IS. The reverse reads has the UMI and viral DNA barcode sequences, enabling identification of the true UMI molecules, linkage of the barcode and IS for a UMI molecule. Part of Figure S3a created in BioRender. Kim, J. (2025) https://BioRender.com/x62k792

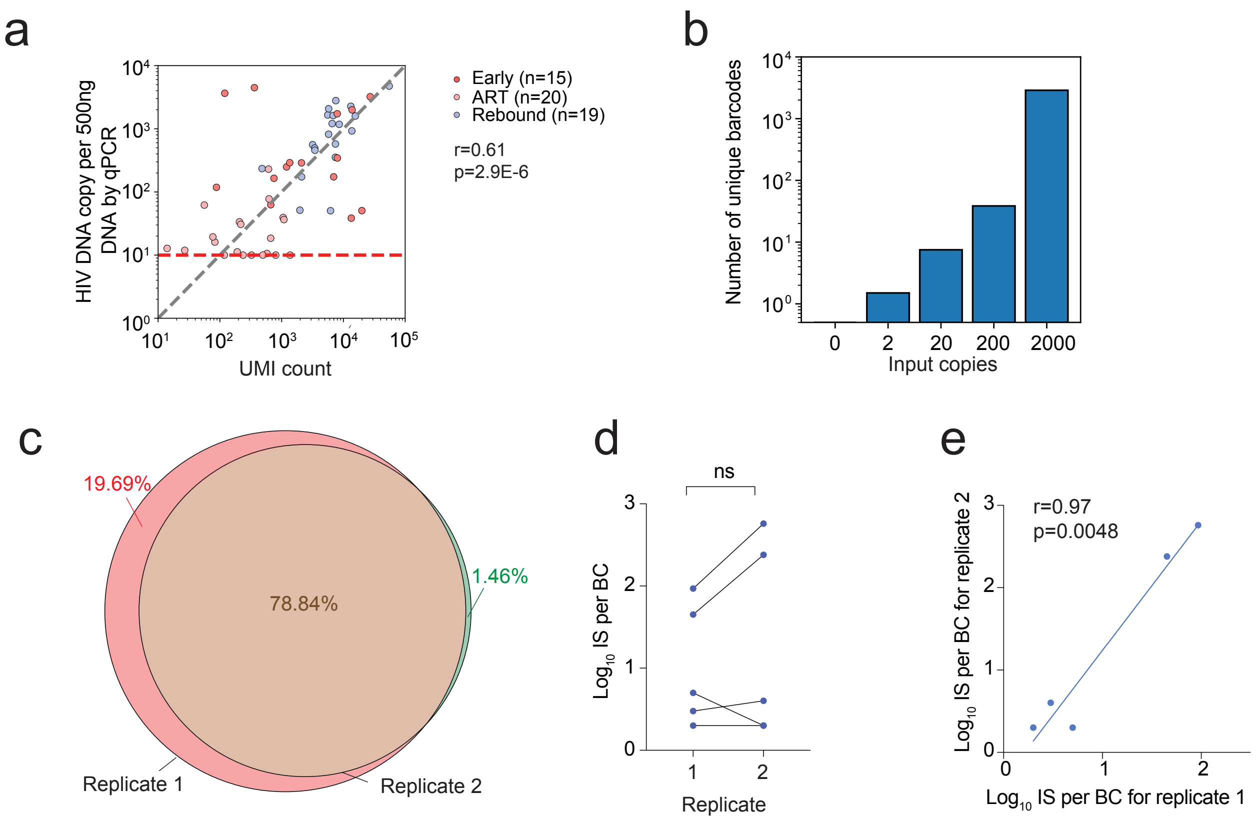

Fig. S4: Barcode detection via BI-seq was sensitive and reproducible.
a, We also performed serial dilutions of HIV DNA copies into uninfected mouse genomic DNA and quantified the DNA using BI-seq. Correlation of DNA viral loads quantified as UMI of DNA molecules by BI-seq or HIV DNA copies by qPCR method for each organ. r, Spearman's correlation coefficient with associated *p* value. The red dashed line indicates the detection limit of 10 copies per reaction by qPCR.
b, The detection limit of BI-seq was determined. The number of plasmid DNA copies of NFNSX-BC was quantified by digital PCR. Then, varying input number of NFNSX-BC plasmid DNA molecules were mixed with uninfected C57/B6 mouse genomic DNA. Because the barcode library has a complexity of >100,000, the possibility of having two plasmid molecule with identical barcode was negligible. The number of barcodes recovered from the sequencing data was 15%-50%.
c-e, Barcode composition from two replicates sampling the same organ. c) Weighted Venn diagram showing the overlapping of pooled barcodes in these two replicates. The number of proviral barcodes between the replicates showed a strong and significant correlation with a Pearson correlation coefficient of 0.97 (p = 0.0048). d) Among the five overlapping proviral barcodes, the IS per BC for each barcode in replicates 1 and 2 were similar. *P* value was calculated by paired t-test. e) IS per BC for each barcode in replicate 1 (x axis) and replicate 2 (y axis) were highly correlated. r represents Pearson's correlation coefficient with associated p value.

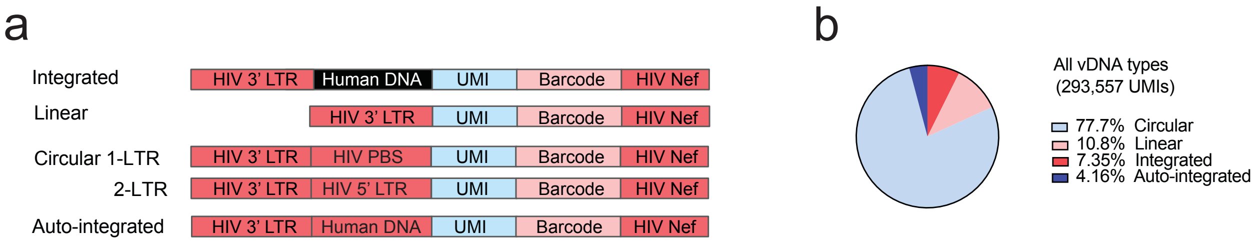

Provirus Type	DNA sequence
Integrated	Mapped to reference human genome (hg38)
Linear	Mapped to <10 bp
Circular	Mapped to region immediately downstream of HIV 3' LTR
Auto-integrated	Mapped to HIV genome, but not circular

Fig. S5: Viral DNA types detected by BI-seq.
a, Schematic showing how integrated, linear, circular and auto-integrated viral DNA types were classified according to the sequence attached to the UMI.
b, Contribution of viral DNA (vDNA) types by UMI to all vDNA types among 60 infected organs from 20 infected animals.

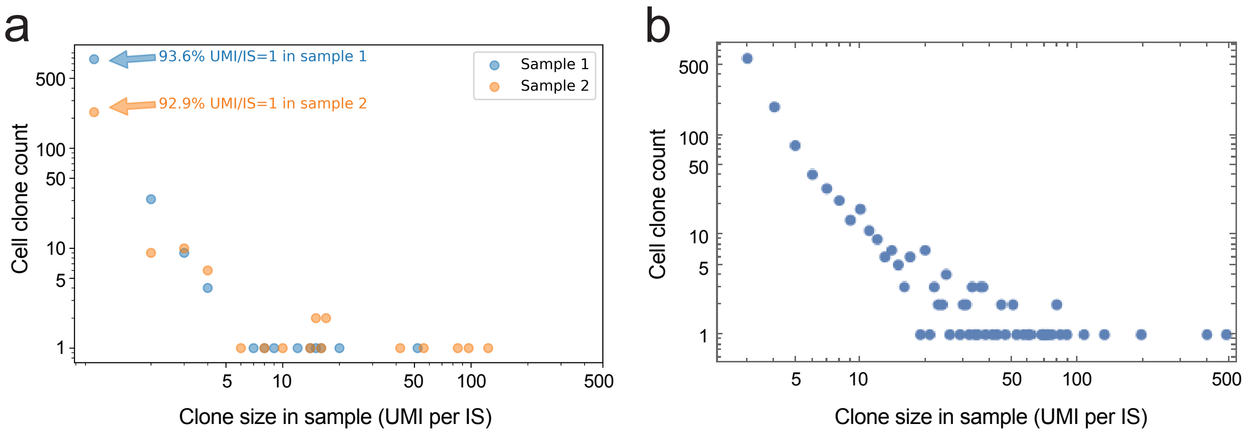

Fig. S6: The distribution of UMI per IS.
a, The UMI per IS distribution in the two replicates from Figure S4c-3. Similar to our simulation and as described in the literature the proviral clone size (UMI/IS) follows a power-law distribution[1]. The non-proliferative clone size was measured to be 93.6% in one sample, and 92.9% in the other sample.
b, The distribution of UMI per IS measurements in all the 48 experiments (combined). Here we assume the clone size of proliferative CD4 T cells obey a power-law distribution with the power $\alpha = 1$[1]. Our simulation shows the distribution of the UMI per IS, which we measured and reflects the clonal composition of the organs.

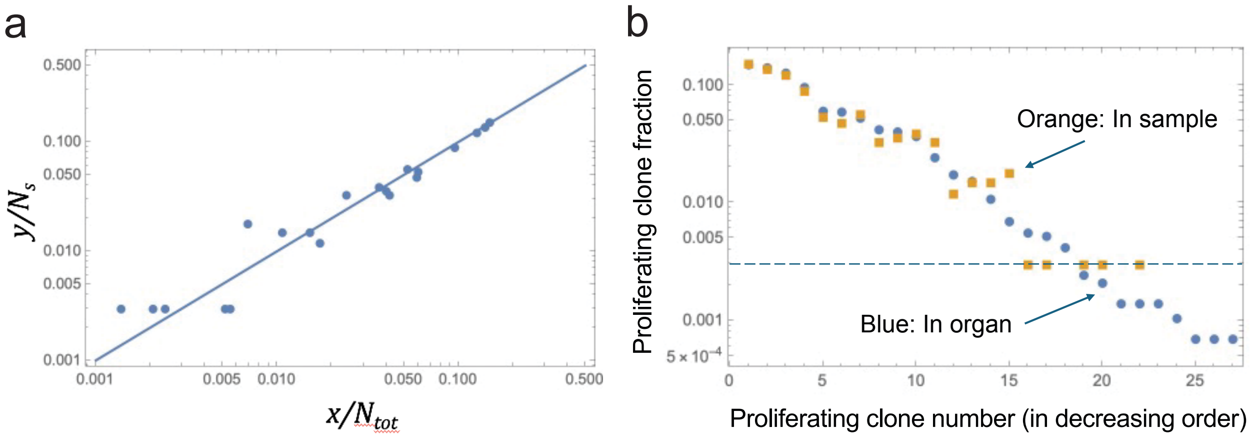

Fig. S7: Simulated sampling experiment, a typical run from spleen data during ART.
a, Predicted (y_i/N_s) clone fractions vs "true" (x_i/N_{tot}) clone fractions for proliferative cells. The diagonal line is $y_i/N_s = x_i/N_{tot}$, a perfect prediction.
b, All the simulated clones sorted by size. The true clone fraction (blue) and the predicted clone fraction (orange) are plotted for each clone. The thin dashed line corresponds to the cases where only a single cell was present in the sample. Parameters are $N_{tot} = 2900$, $N_{prol} = N_{non} = N_{tot}/2$, $N_s = 339$, $\alpha = 1$.

a

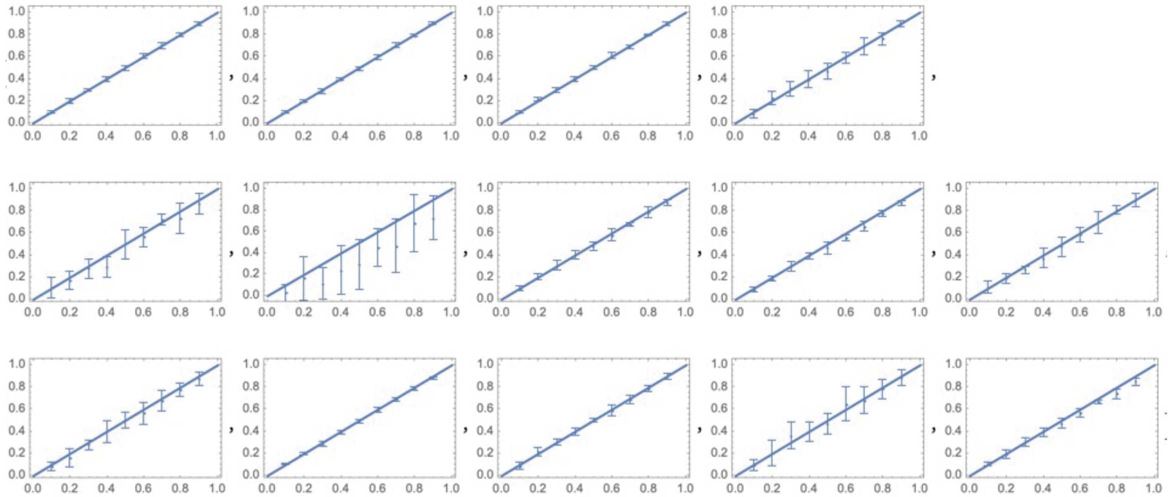

b

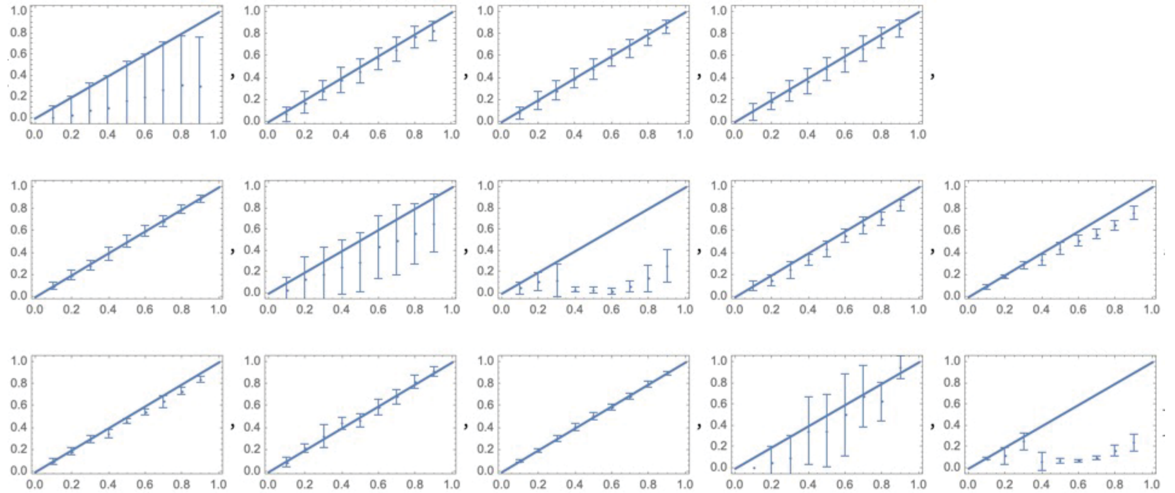

c

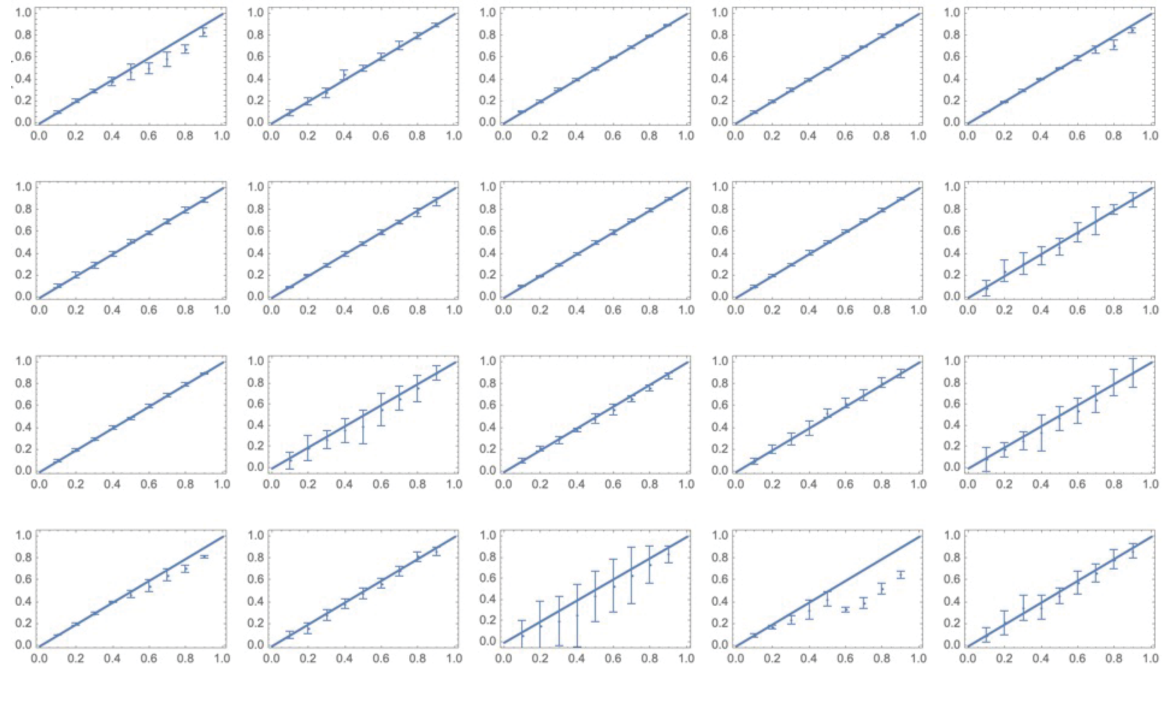

Fig. S8: The predicted vs “true” percentage of proliferating cells in the tissue using values (N_{tot}, N_s) from infected samples.

Each panel corresponds to a single pair (N_{tot}, N_s). The horizontal axis in each graph is ν (true fraction) and the vertical axis is $\nu^{(s)}$ (predicted fraction). The symbols with vertical bars represent means and standard derivation from 100 simulations. The straight line is $\nu^{(s)} = \nu$, which is a perfect match. Results for the parameters corresponding to three timepoints of the experiment: early infection (a), ART (b), and rebound infection (c), respectively.

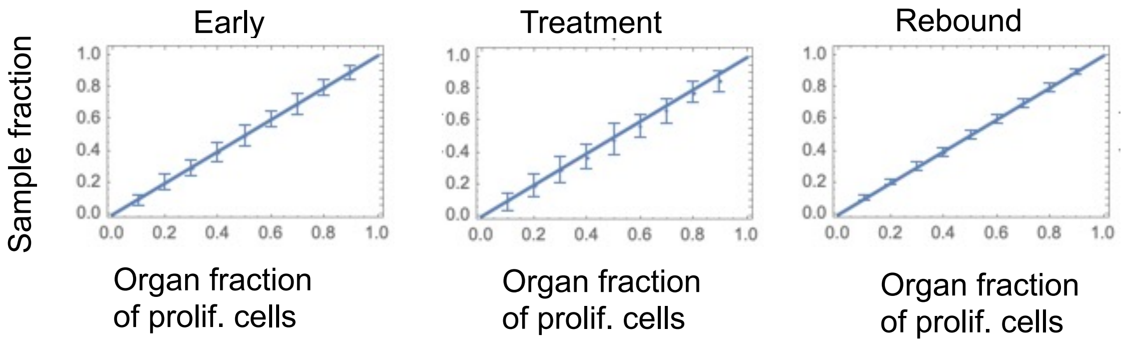

Fig. S9: A summary of the predicted vs "true" percentage of proliferating cells in the tissue. Average values of (N_{tot}, N_s) from samples during early infection, ART, and rebound infection are plotted. The horizontal axis in each graph is ν (true fraction) and the vertical axis is $\nu^{(s)}$ (predicted fraction). The symbols with vertical bars represent means and standard derivation from 100 simulations. The straight line is $\nu^{(s)} = \nu$.

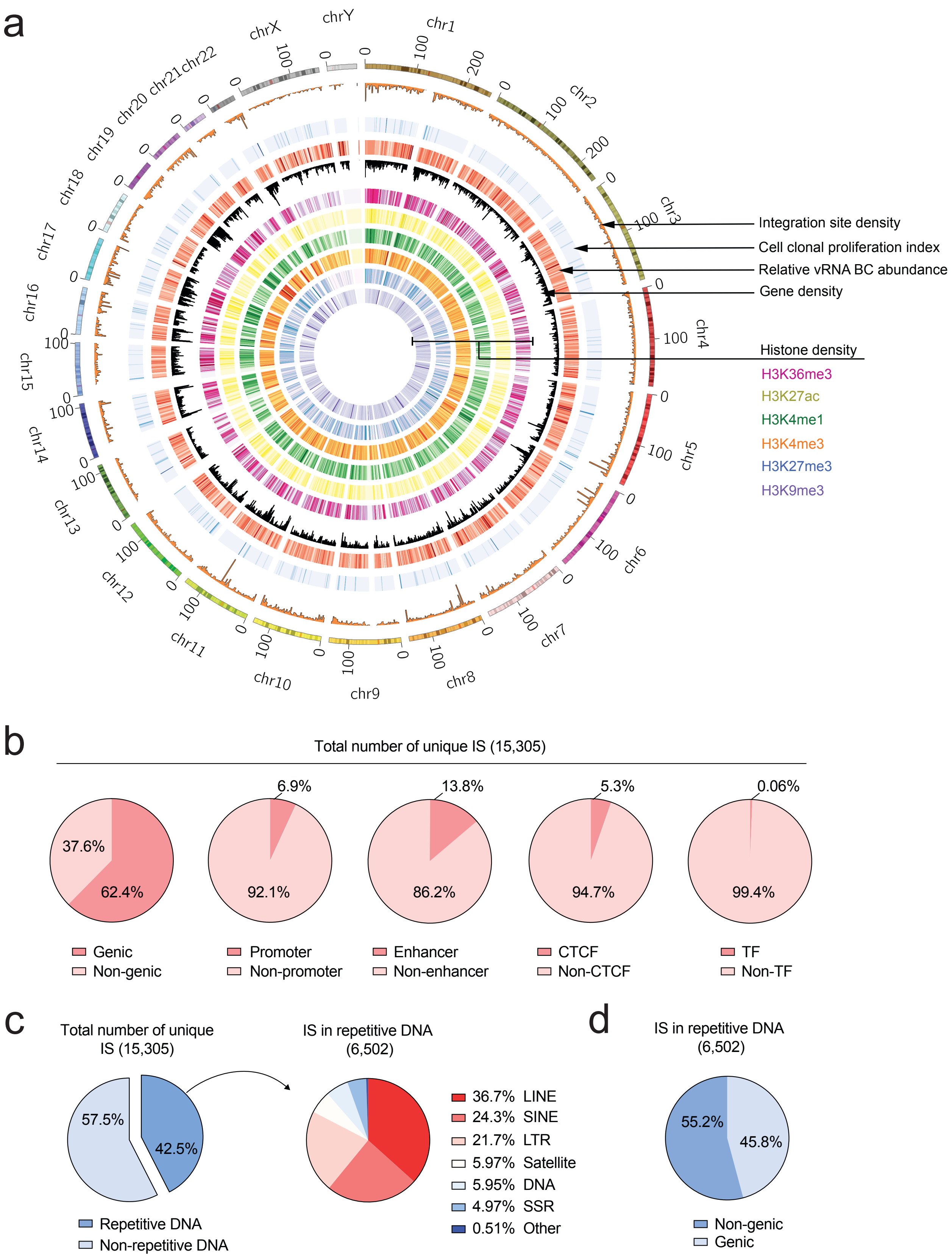
a
chrX
chrY
chr1
chr2
chr3
chr4
chr5
chr6
chr7
chr8
chr9
chr10
chr11
chr12
chr13
chr14
chr15
chr16
chr17
chr18
chr19
chr20
chr21
chr22
Integration site density
Cell clonal proliferation index
Relative vRNA BC abundance
Gene density
Histone density
H3K36me3
H3K27ac
H3K4me1
H3K4me3
H3K27me3
H3K9me3
b
Total number of unique IS (15,305)
37.6%
62.4%
6.9%
92.1%
13.8%
86.2%
5.3%
94.7%
0.06%
99.4%
Genic
Non-genic
Promoter
Non-promoter
Enhancer
Non-enhancer
CTCF
Non-CTCF
TF
Non-TF
c
Total number of unique IS (15,305)
57.5%
42.5%
Repetitive DNA
Non-repetitive DNA
IS in repetitive DNA (6,502)
36.7% LINE
24.3% SINE
21.7% LTR
5.97% Satellite
5.95% DNA
4.97% SSR
0.51% Other
d
IS in repetitive DNA (6,502)
55.2%
45.8%
Non-genic
Genic

Fig. S10: Integration sites enriched in genes and non-repetitive DNA.
a, Circos plot showing the integration site density, cell clone size, relative vRNA BC abundance, gene density, and histone modification density
b, Contribution of proviruses integrated in genes or other gene regulatory elements such as promoters, enhancers, CCCTC-binding factor “CTCF” binding sites, and transcription factor “TF” binding sites.
c, Contribution of proviruses integrated in repetitive and non-repetitive DNA elements (left). Among the proviruses in repetitive DNA elements, the proportion in LINE, SINE, LTRs, which include retroposons, satellite repeats, DNA repeat elements “DNA”, simple-sequence repeats “SSR” or micro-satellites, or other (e.g. low complexity repeats, RNA repeats, or repeats including rolling circle, or unknown).
d, Among the proviruses integrated in repetitive DNA elements, the proportion that are located in genic or non-genic DNA regions.

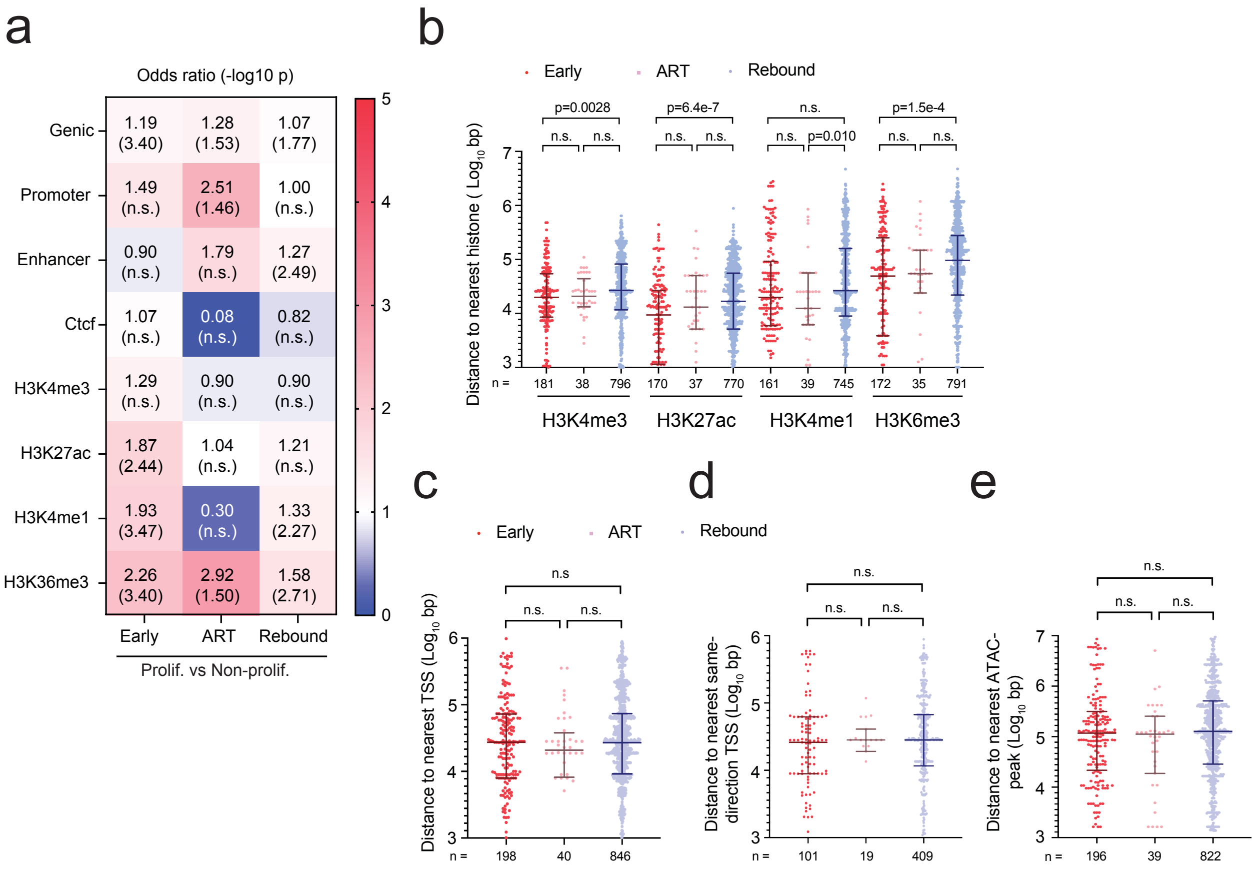

Fig. S11: Proviruses in proliferated cell clones have some persistent activating chromatin features over time.

a, Heat map demonstrating the odds ratio of the listed genetic and epigenetic features of proviruses from proliferated versus non-proliferated cell clones by timepoint. The odds ratio and p values were calculated using the Fisher's exact test.

b, Chromosomal distance to the nearest histone modification among proviruses from proliferated cell clones during acute infection, ART suppression, or rebound infection.

c-e, Chromosomal distance to the nearest TSS (c), same-direction TSS (d), and ATAC-peak (e) among proviruses from proliferated cell clones during acute infection, ART, or rebound infection. Horizontal bars represent mean ± SD. P values were calculated using the two-tailed Mann-Whitney test (b-e).

a

HIV-1-Infected GHOST (3) CXCR4$^+$CCR5$^+$ cells

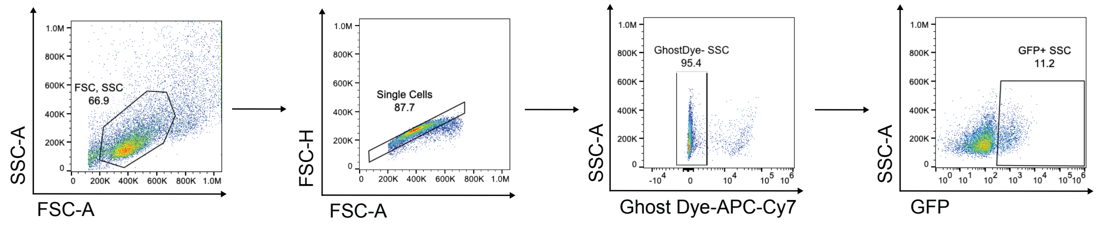

b

Human immune cells from BLT mice

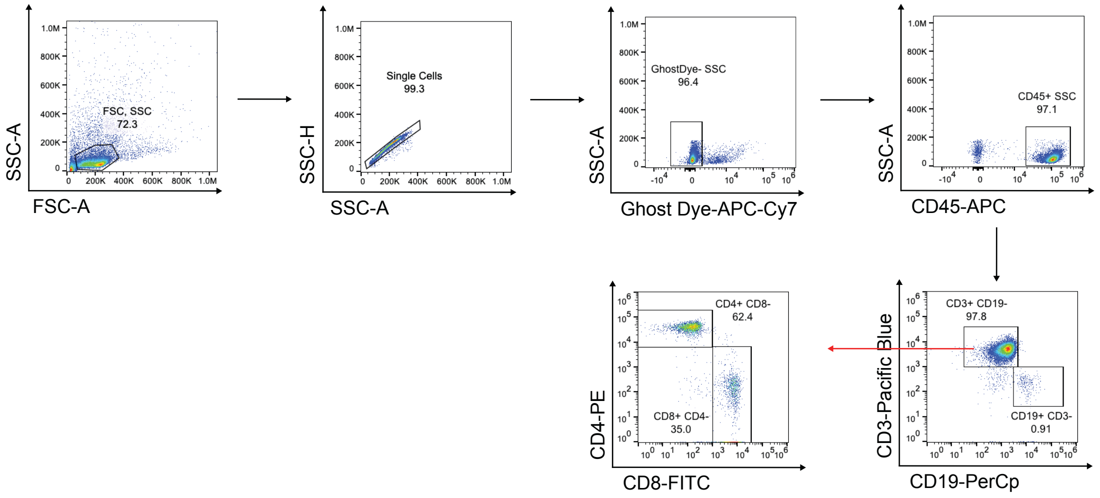

Fig. S12: Flow cytometry gating strategies.
a, b, Flow cytometric analysis of HIV-infected Ghost CXCR4+CCR5+ cells in Figure S1f (a) and human immune cells from BLT mice in Figure S2a (b).

Supplementary Note 1. Mathematical analysis of the sampling procedure

The dataset consist of 48 different cases corresponding to different animals, different organs, and different timepoints. While we do not have information on the clone size of proliferating cells in different organs, we have $\mathcal{N}_{tot}$, which is the number of all live cells in an organ, $\mathcal{N}_s$, which is the number of all cells that were used to count the number of CD4 T cells containing provirus (for each experiment), and N_s, which is the obtained count of CD4 T cells containing the provirus in each sample. Note that the total number of live cells in the organ and in the sample ($\mathcal{N}_{tot}$ and $\mathcal{N}_s$) are much larger that the corresponding number of cells containing a provirus (N_{tot} and N_s). Assuming a well-mixed population, we will postulate that the proportion of CD4 T cells containing provirus is the same in the whole organ and in the sample that was analyzed. Therefore, we can calculate

$$N_{tot} = N_s \frac{\mathcal{N}_{tot}}{\mathcal{N}_s}.$$

In order to calculate the error in the estimates for the percentage of proliferating cells in each experiment, we developed the following numerical procedure. For each of the experiments, the quantities N_{tot} and N_s are given. While we do not know the true fraction of proliferating cells in each organ, we will view this as a variable, $\nu \in [0, 1]$, such that the number of

proliferating and non-proliferating cells is given by

$$N_{prol} = \nu N_{tot}, \qquad N_{non} = (1 - \nu) N_{tot},$$

respectively. We will assume that the clone sizes of proliferative CD4 T cells obey a power-law distribution:

$$g_n = Cn^{-\alpha}, \;\; n = 2, 3, \ldots, N_{tot},$$

with the power $\alpha = 1$ (see supplemental reference[1]), and C is the normalization constant. This is consistent with the distribution of the UMI per IS, which we measure and which reflects the clonal composition of the organs, see Figure S6.

For a given fraction of proliferating cells, ν, we can create the clone sizes $x_1, x_2, \ldots, x_m$ of proliferating cells that are drawn from the above distribution, such that the total number of these cells equals $\sum_{i=1}^{m} x_i = N_{prol}$. Non-proliferating cells can be viewed as clones of size 1: $x_i = 1$ for $i = m + 1, \ldots, m + N_{non}$.

After sampling N_s times, we can obtain the values $y_1, y_2, \ldots, y_{m+N_{non}}$ that correspond to the number of times each clone was represented in the sample, with $0 \le y_i \le \min\{x_i, N_s\}$. Note that non-proliferating cells can only be represented zero or one time in the sample. Let us denote the number of clones represented exactly once ($y_i = 1$) by $N_{non}^{(s)}$ and the number of clones represented more than once by $N_{prol}^{(s)}$. The estimate for the fraction of proliferating cells is then given by

$$\nu^{(s)} = \frac{N_{prol}^{(s)}}{N_s}.$$

A typical result of a simulation is shown in Figure S7. Figure S7a shows predicted (y_i/N_s) clone fractions vs "true" (x_i/N_{tot}) clone fractions for proliferative cells. The diagonal line is $y_i/N_s = x_i/N_{tot}$, a perfect prediction. Figure S7b sorts all the clones by size and shows (in blue) the true clone fraction and (in orange) the predicted clone fraction. The thin dashed line corresponds to the cases where only a single cell was present in the sample. As expected, the clones that show up as "non-proliferative" (a single cell in the sample) tend to be smaller in size.

Figure S8 shows results of such simulations, where we used 9 values of the true (but unknown) fraction of proliferating cells (the horizontal axis): $\nu \in \{0.1, 0.2, \dots, 0.9\}$, and used 100 simulations per point to calculate the predicted $\nu^{(s)}$ value (the vertical axis; means and standard deviations are shown). A perfect match is shown by straight lines $\nu^{(s)} = \nu$. Figure S8 shows results for the parameters corresponding to three timepoints of the experiment, early infection (Fig. S8a), ART (Fig. S8b), and rebound infection (Fig. S8c), respectively.

Figure S9 presents summary simulations for the cases of early infection, ART, and rebound infection. The probability to predict the fraction of proliferating cells within 10% is given by, on average, 91%, 76%, and 99% during early infection, ART, and rebound infection respectively. The difference between the mean prediction and the true fraction of proliferative cells is given by 0.8%, 2.3%, and 0.2% in the three timepoints respectively.

In conclusion, our method gives reliable predictions for the fraction of proliferative cells for most of the 48 experiments. The small number of poorer predictions correspond to the cases where the sample size is about 1% or less of the total cell number. The reason the method works is because even though some clones are misclassified as non-proliferative, they are typically smaller clones and comprise a small fraction of the proliferating cell population.

Supplemental References

1 Gaimann, M. U., Nguyen, M., Desponds, J. & Mayer, A. Early life imprints the hierarchy of T cell clone sizes. *Elife* **9** (2020). https://doi.org:10.7554/eLife.61639
