# Supplementary material for: Barcoded HIV-1 reveals viral persistence driven by clonal proliferation and distinct epigenetic patterns
Source: Nat Commun. 2025 Feb 14;16:1641. doi: 10.1038/s41467-025-56771-4 (PMC11829055; doi:10.1038/s41467-025-56771-4)
Supplement: Supplementary file 2 — Description of Additional Supplementary Files [file 41467_2025_56771_MOESM2_ESM.docx]

Description of Additional Supplementary Files

File Name: Supplementary Data 1. Viral RNA barcodes in vivo and the UMI per barcode (BC)
Description: Viral RNA barcode (BC) sequences were classified by mouse, organ, timepoint, and UMI per BC.

File Name: Supplementary Data 2. Proviral barcodes (BC) in vivo

Description: Proviral barcodes (BC) in vivo were classified by mouse, organ, timepoint and the UMI per BC, integration site (IS) per BC, UMI per BC (normalized to all BC in that sample), and UMI per IS, and the abundance of that viral BC in the plasma among all the BC variants in that sample.

File Name: Supplementary Data 3. Number of live cells and frequency of CD4+ T cells
Description: Number of live cells from each organ (spleen, bone marrow, thymic implant) were reported for each animal. Number of live cells used for BI-seq and frequency of CD4+ T cells per sample were also reported.

File Name: Supplementary Data 4. Integration sites (IS) and linked proviral barcode (BC) in vivo
Description: IS and linked proviral BC were listed and classified by mouse, organ, and timepoint. IS were aligned to the hg38 Ensemble release 108 and genetic annotations reported for chromosomal location, host gene and ENSG identifiers, circos bin, and chromosomal distance (bp) to nearby genomic features such as genes, promoter, enhancer, CCCTC-binding factor (CTCF) binding site, and transcription (TF) binding site. IS were also aligned to chromatin immunoprecipitation sequencing (CHIP-seq) deep sequencing data of resting primary CD4 T cells histone modifications in the Encyclopedia of DNA element (ENCODE) regions and nearest chromosomal distance (bp) to histone modifications HEK27me3, H3K9me3, H3K4me3, H3K27ac, H3K4me1, and H3K36me3), transcriptional start site (TSS), same-orientation TSS, and nearest ATAC-peak were reported. Integration sites were compared to the list of genes associated with cancer based on the Catalogue of Somatic Mutations in Cancer (COSMIC) Cancer Gene Census (CGC). Transcription frequency of the viral barcode in the plasma was also reported for each proviral barcode.

File Name: Supplementary Data 5. Integration events that were enriched in genes among proviruses associated with or without viremia and viral reseeding
Description: The host gene and ENSG identifiers associated with integration events were listed. The number of integration events enriched in genes among proviruses associated with or without viremia were compared. Odds ratio and p values were calculated by two-sided Fisher’s exact test.

File Name: Supplementary Data 6. List of primers used in this study
Description: List of primers in this study classified by name, primer sequence, and commercial vendor from which they were purchased.
