# Supplementary material for: Barcoded HIV-1 reveals viral persistence driven by clonal proliferation and distinct epigenetic patterns
Source: Nat Commun. 2025 Feb 14;16:1641. doi: 10.1038/s41467-025-56771-4 (PMC11829055; doi:10.1038/s41467-025-56771-4)
Supplement: Supplementary file 4 — Reporting Summary [file 41467_2025_56771_MOESM4_ESM.pdf]

nature portfolio

Corresponding author(s): Jocelyn Kim

Last updated by author(s): January 15, 2025

Reporting Summary

Nature Portfolio wishes to improve the reproducibility of the work that we publish. This form provides structure for consistency and transparency in reporting. For further information on Nature Portfolio policies, see our Editorial Policies and the Editorial Policy Checklist.

Statistics

For all statistical analyses, confirm that the following items are present in the figure legend, table legend, main text, or Methods section.

n/a	Confirmed	
☐	☒	The exact sample size (*n*) for each experimental group/condition, given as a discrete number and unit of measurement
☐	☒	A statement on whether measurements were taken from distinct samples or whether the same sample was measured repeatedly
☐	☒	The statistical test(s) used AND whether they are one- or two-sided<br>*Only common tests should be described solely by name; describe more complex techniques in the Methods section.*
☒	☐	A description of all covariates tested
☒	☐	A description of any assumptions or corrections, such as tests of normality and adjustment for multiple comparisons
☐	☒	A full description of the statistical parameters including central tendency (e.g. means) or other basic estimates (e.g. regression coefficient) AND variation (e.g. standard deviation) or associated estimates of uncertainty (e.g. confidence intervals)
☐	☒	For null hypothesis testing, the test statistic (e.g. *F*, *t*, *r*) with confidence intervals, effect sizes, degrees of freedom and *P* value noted<br>*Give P values as exact values whenever suitable.*
☒	☐	For Bayesian analysis, information on the choice of priors and Markov chain Monte Carlo settings
☒	☐	For hierarchical and complex designs, identification of the appropriate level for tests and full reporting of outcomes
☐	☒	Estimates of effect sizes (e.g. Cohen's *d*, Pearson's *r*), indicating how they were calculated

Our web collection on statistics for biologists contains articles on many of the points above.

Software and code

Policy information about availability of computer code

Data collection	Deep sequencing was performed on HiSeq3000 platform (Illumina) and NovaSeq6000 PE150 sequencing with PE150 settings.
Data analysis	Deep sequencing analysis was performed using Bowtie2 (v2.4.2), Jupyter (v7.2.1), Bedtools (v2.31.1), SciPy (1.13.1), and BioPython (v1.83).

For manuscripts utilizing custom algorithms or software that are central to the research but not yet described in published literature, software must be made available to editors and reviewers. We strongly encourage code deposition in a community repository (e.g. GitHub). See the Nature Portfolio guidelines for submitting code & software for further information.

Data

Policy information about availability of data

All manuscripts must include a data availability statement. This statement should provide the following information, where applicable:

- Accession codes, unique identifiers, or web links for publicly available datasets
- A description of any restrictions on data availability
- For clinical datasets or third party data, please ensure that the statement adheres to our policy

All original codes have been downloaded onto github at https://doi.org/10.5281/zenodo.5723824.. Raw sequencing reads have been deposited on NCBI SRA (Sequencing Read Archive) database PRJNA963079.

Research involving human participants, their data, or biological material

Policy information about studies with human participants or human data. See also policy information about sex, gender (identity/presentation), and sexual orientation and race, ethnicity and racism.

Reporting on sex and gender	*Use the terms sex (biological attribute) and gender (shaped by social and cultural circumstances) carefully in order to avoid confusing both terms. Indicate if findings apply to only one sex or gender; describe whether sex and gender were considered in study design; whether sex and/or gender was determined based on self-reporting or assigned and methods used. Provide in the source data disaggregated sex and gender data, where this information has been collected, and if consent has been obtained for sharing of individual-level data; provide overall numbers in this Reporting Summary. Please state if this information has not been collected. Report sex- and gender-based analyses where performed, justify reasons for lack of sex- and gender-based analysis.*
Reporting on race, ethnicity, or other socially relevant groupings	*Please specify the socially constructed or socially relevant categorization variable(s) used in your manuscript and explain why they were used. Please note that such variables should not be used as proxies for other socially constructed/relevant variables (for example, race or ethnicity should not be used as a proxy for socioeconomic status). Provide clear definitions of the relevant terms used, how they were provided (by the participants/respondents, the researchers, or third parties), and the method(s) used to classify people into the different categories (e.g. self-report, census or administrative data, social media data, etc.) Please provide details about how you controlled for confounding variables in your analyses.*
Population characteristics	*Describe the covariate-relevant population characteristics of the human research participants (e.g. age, genotypic information, past and current diagnosis and treatment categories). If you filled out the behavioural & social sciences study design questions and have nothing to add here, write "See above."*
Recruitment	*Describe how participants were recruited. Outline any potential self-selection bias or other biases that may be present and how these are likely to impact results.*
Ethics oversight	*Identify the organization(s) that approved the study protocol.*

Note that full information on the approval of the study protocol must also be provided in the manuscript.

Field-specific reporting

Please select the one below that is the best fit for your research. If you are not sure, read the appropriate sections before making your selection.

☒ Life sciences ☐ Behavioural & social sciences ☐ Ecological, evolutionary & environmental sciences

For a reference copy of the document with all sections, see nature.com/documents/nr-reporting-summary-flat.pdf

Life sciences study design

All studies must disclose on these points even when the disclosure is negative.

Sample size	Experimental replicates and group sizes were based on our prior experience with each of the model systems used. Groups of 5-15 animals per condition have historically proven sufficient to discern differences in virus replication profile in these and similar models.
Data exclusions	Only HIV-infected animals with undetectable plasma viral loads at the end of ART treatment and no graft-versus-host disease were included in analysis.This was pre-defined because these experimental factors would confound study of HIV latency and rebound.
Replication	In vitro HIV replication experiments consist of at least 3 independent experiments as indicated in the figure legends. The number of independent experiments performed is in the legends of the manuscript figures. We confirm all attempts at replication were successful.
Randomization	Male and female animals were similarly distributed among groups. Mouse groups were randomized to ensure similar pre-ART viral loads between groups.
Blinding	Investigators were not blinded because the groups of animals were sacrificed at set timepoints. However, data and sequence analysis was conducted in a blinded fashion.

Reporting for specific materials, systems and methods

We require information from authors about some types of materials, experimental systems and methods used in many studies. Here, indicate whether each material, system or method listed is relevant to your study. If you are not sure if a list item applies to your research, read the appropriate section before selecting a response.

Materials & experimental systems

n/a	Involved in the study	
☐	☒	Antibodies
☐	☒	Eukaryotic cell lines
☒	☐	Palaeontology and archaeology
☐	☒	Animals and other organisms
☒	☐	Clinical data
☒	☐	Dual use research of concern
☒	☐	Plants

Methods

n/a	Involved in the study	
☒	☐	ChIP-seq
☐	☒	Flow cytometry
☒	☐	MRI-based neuroimaging

Antibodies

Antibodies used	Cells from animals were stained with fluorescently conjugated antibodies: CD14-Brilliant Violet 510 (Biolegend, clone M5E2, #301842, 5 µl per million cells), CD3-Pacific Blue (Biolegend, clone Hit3a, #300330, 2.0 µg per million cells), CD8a-FITC (Biolegend, clone Hit8a, #300906, 5 µl per million cells), CD4-PE (Biolegend, clone RPA-T4, #300508, 5 µl per million cells), CD19-PE-Cy5 (Biolegend, clone SJ25C1, #363042, 5 µl per million cells), CD45-APC (Biolegend, clone 2D1, #368512, 5 µl per million cells), and CD56-PE-Cy7 (Biolegend, clone MEM-188, #304628, 5 µl per million cells).
Validation	In addition to the standard antibody validation that occurs at the commercial vendor, we validate each antibody and compare to isotype staining.

Eukaryotic cell lines

Policy information about cell lines and Sex and Gender in Research

Cell line source(s)	GHOST (3) CXCR4+CCR5+ cells were obtained through the NIH AIDS Reagent Program, Division of AIDS, NIAID, NIH, from Dr. Vineet N. KewalRamani and Dr. Dan R. Littman (cat# 3942). 293T cells were obtained from ATCC (ATCC CRL-11268).
Authentication	Obtained directly from NIH AIDS Reagent repository or ATCC. Phenotype and characteristics are as expected therefore they were not subjected to further authentication procedures.
Mycoplasma contamination	All cell lines tested negative for mycoplasma contamination.
Commonly misidentified lines (See ICLAC register)	None used in this study.

Animals and other research organisms

Policy information about studies involving animals; ARRIVE guidelines recommended for reporting animal research, and Sex and Gender in Research

Laboratory animals	B6.129S-Rag2tm1Fwa Cd47tm1Fpl Il2rgtm1Wjl/J from the Jackson Laboratory. Male (n=8) and female (n=12) mice were age-matched and between 6 and 8 weeks old. Mice were housed in a 12h light/12h dark cycle at temperatures between 18-23 Celsius and 40-60% humidity.
Wild animals	None
Reporting on sex	Sex was not included in the analysis of this study due to constraints in sample size. While sex may play role, this was outside the scope of the current investigation.
Field-collected samples	None
Ethics oversight	All mouse experiments were performed in ethical compliance with the study protocol approved by the UCLA Animal Research Committee (ARC-2021-020).

Note that full information on the approval of the study protocol must also be provided in the manuscript.

Plants

Seed stocks

Report on the source of all seed stocks or other plant material used. If applicable, state the seed stock centre and catalogue number. If plant specimens were collected from the field, describe the collection location, date and sampling procedures.

Novel plant genotypes

Describe the methods by which all novel plant genotypes were produced. This includes those generated by transgenic approaches, gene editing, chemical/radiation-based mutagenesis and hybridization. For transgenic lines, describe the transformation method, the number of independent lines analyzed and the generation upon which experiments were performed. For gene-edited lines, describe the editor used, the endogenous sequence targeted for editing, the targeting guide RNA sequence (if applicable) and how the editor was applied.

Authentication

Describe any authentication procedures for each seed stock used or novel genotype generated. Describe any experiments used to assess the effect of a mutation and, where applicable, how potential secondary effects (e.g. second site T-DNA insertions, mosiacism, off-target gene editing) were examined.

Flow Cytometry

Plots

Confirm that:

- [x] The axis labels state the marker and fluorochrome used (e.g. CD4-FITC).
- [x] The axis scales are clearly visible. Include numbers along axes only for bottom left plot of group (a 'group' is an analysis of identical markers).
- [x] All plots are contour plots with outliers or pseudocolor plots.
- [x] A numerical value for number of cells or percentage (with statistics) is provided.

Methodology

Sample preparation

Cells are either from human peripheral blood mononuclear cells from the UCLA virology core, GHOST (3) CXCR4+CCR5+ cells, which upon HIV infection and viral tat protein expression, express GFP, or single cell suspensions isolated from mouse tissues. During PBMC staining 1E5 to 1E6 cells were suspended in a 50ul volume of 1:1 dilution of PBS: human Ab serum (Sigma). For 1-step staining to detect surface markers, cells were stained with FACS antibodies at 4C for 20min and then after washed. Cells were fixed in 4% paraformaldehyde in PBS. Cells were stored in PBS and 0.5% BSA prior to running on a MACSQuant Analyzer 10 flow cytometer (Miltenyi) or Attune NxT (Beckman Coulter).

Instrument

MACSQuant Analyzer 10 flow cytometer (Miltenyi) or Attune NxT (Beckman Coulter)

Software

FlowJo 10.6.0 (TreeStart, Inc)

Cell population abundance

No fluorescent activated cell sorting was performed in these studies.

Gating strategy

Describe the gating strategy used for all relevant experiments, specifying the preliminary FSC/SSC gates of the starting cell population, indicating where boundaries between "positive" and "negative" staining cell populations are defined.

- [x] Tick this box to confirm that a figure exemplifying the gating strategy is provided in the Supplementary Information.
